# Stereospecificity of hydride transfer and molecular docking in FMN‐dependent NADH‐indigo reductase of *Bacillus smithii*

**DOI:** 10.1002/2211-5463.13200

**Published:** 2021-06-15

**Authors:** Kazunari Yoneda, Haruhiko Sakuraba, Tomohiro Araki, Toshihisa Ohshima

**Affiliations:** ^1^ Department of Bioscience School of Agriculture Tokai University Kumamoto Japan; ^2^ Department of Applied Biological Science Faculty of Agriculture Kagawa University Kita‐gun Japan; ^3^ Department of Biomedical Engineering Faculty of Engineering Osaka Institute of Technology Japan

**Keywords:** H‐NMR, cofactor stereospecificity, FMN‐dependent NADH‐indigo reductase, molecular docking simulation

## Abstract

In this study, we investigated the stereospecificity of hydride transfer from NADH to flavin mononucleotide (FMN) in reactions catalyzed by the FMN‐dependent NADH‐indigo reductase expressed by thermophilic *Bacillus smithii*. We performed ^1^H‐NMR spectroscopy using deuterium‐labeled NADH (4*R*‐^2^H‐NADH) and molecular docking simulations to reveal that the pro‐*S* hydrogen at the C4 position of the nicotinamide moiety in NADH was specifically transferred to the flavin‐N5 atom of FNM. Altogether, our findings may aid in the improvement of the indigo dyeing (Aizome) process.

## Graphical abstract

The stereospecificity of hydride transfer from NADH to flavin mononucleotide (FMN) in reactions catalyzed by the FMN‐dependent NADH‐indigo reductase from the thermophilic bacterium *Bacillus smithii* was studied. Both analyses of ^1^H‐NMR spectroscopy using deuterium‐labeled NADH (4*R*‐^2^H‐NADH) and molecular docking simulations showed that the pro‐*S* hydrogen of NADH was specifically transferred to the flavin‐N5 atom of FNM.

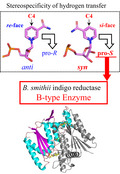

**Abbreviations**
FMN: flavin mononucleotide
PDB: Protein Data Bank

Indigo reductase expressed by *Bacillus* sp. AO1 is a flavin mononucleotide (FMN)‐dependent NADH‐azoreductase (EC 1.7.1.6) that catalyzes the reductive cleavage of azo groups (R–N=N–R) in aromatic azo compounds and the reduction of indigo compounds (lacking an azo group) [1]. Three‐dimensional structural information and enzymological characteristics of azoreductases from several microbial species have already been reported [2, 3, 4, 5, 6, 7]. However, the structure of the NAD(H)‐azoreductase complex and the mechanism of transfer of hydrogen from NADH are unknown. Although the crystal structure of *Bacillus* sp. B29 azoreductase with Cibacron blue as one of the substrates has been reported, the mechanism of NADP binding to the active site cannot be elucidated in that structure [7]. Generally, NAD(P)H‐dependent dehydrogenases show either pro*‐R* or pro*‐S* stereospecificity for hydrogen transfer from the C4 position of the nicotinamide moiety in NAD(P)H [8, 9]. The difference in mechanism of the hydride transfer is known to result in distinct differences in the structure and function of NAD(P)H‐dependent enzyme groups [10]. The stereospecificity of azoreductases during electron transfer between NADH and FMN is relatively unknown, except for that of FMN‐dependent NAD(P)H‐quinone oxidoreductase [11].

Recently, we reported the crystal structure of thermostable FMN‐dependent indigo reductase expressed by *Bacillus smithii* DSM 4216^T^, which catalyzes the reduction of indigo carmine (water‐soluble indigo) and azo compounds [12]. We also determined the structure‐based stabilization and substrate recognition mechanism of this enzyme [12]. Although we used co‐crystallization and soaking methods for the structural analysis of the indigo reductase expressed by *B. smithii* and NAD(H) complex, we do not know the complex structures of the enzyme with NAD^+^, NADH, and NAD^+^ analogs. In this study, we examined the structure of NAD^+^ bound to the *B. smithii* indigo reductase by using a molecular docking simulation method, and the stereospecificity of hydride transfer from NADH to FMN using ^1^H‐NMR spectroscopy, with 4*R*‐^2^H‐NADH as a cofactor. To our knowledge, this is the first study to elucidate the molecular mechanism of the catalytic reaction of indigo reductase. Determining the stereospecificity of the hydrogen transfer and the structural basis for the catalytic reaction that mediate thermostable indigo reductase may provide useful information for the improvement of the indigo dyeing (Aizome) process.

## Materials and methods

### Estimating the stereospecificity of indigo reductase during hydride transfer from NADH to FMN

4*R*‐^2^H‐NADH was prepared as described previously [13], with a slight modification. Briefly, 5 mL of the reaction mixture containing 4% deuterated ethanol‐d_6_ (CD_3_CD_2_OD; Tokyo Chemical Industry, Tokyo, Japan) was incubated with 225 U of alcohol dehydrogenase (A‐stereospecific; pro‐*R* specific) from *Saccharomyces cerevisiae* (Oriental Yeast, Tokyo, Japan) and 150 μmol of NAD^+^ prepared in 5 mL of 100 mm NH_4_HCO_3_ buffer (pH 7.8) for 20 min at 37 °C. The formation of 4*R*‐^2^H‐NADH was validated by monitoring the increase in absorbance of the reaction mixture at 340 nm (Shimadzu UVmini‐1240 spectrophotometer, Kyoto, Japan). The mixture was then passed through a centrifugal filter (Amicon Ultra 3000 NMWL; Millipore, Billerica, MA, USA) to remove the residual alcohol dehydrogenase. The filtrate was diluted fivefold with Milli‐Q water and injected into a Toyopearl GigaCap DEAE‐650M column (bed volume: 6 mL, Tosoh, Yamaguchi, Japan). 4*R*‐^2^H‐NADH was then eluted using a linear gradient of 0–200 mm NH_4_HCO_3_ buffer (pH 7.8, total 200 mL). Fractions containing 4*R*‐^2^H‐NADH that showed a high absorbance at 340 nm (*A*_260_/*A*_340_ ≤ 2.3) were collected and lyophilized (yield: 6.5 mg of 4*R*‐^2^H‐NADH).

The stereospecificity of hydride transfer from NADH to FMN by the *B. smithii* indigo reductase was analyzed by ^1^H‐NMR spectroscopy using 4*R* deuterium NADH. The expression and purification of recombinant FMN‐bound indigo reductase were carried out as previously described [12].

To determine the stereospecificity of hydrogen transfer from NADH catalyzed by the indigo reductase, a reaction mixture (1.0 mL) containing 5.5 mg of FMN‐bound *B. smithii* indigo reductase and 2 mg of 4*R*‐^2^H‐NADH in 2 mm Tris–HCl buffer (pH 8.0) was incubated for 1 h at 25 °C. Thereafter, the reaction mixture was passed through a centrifugal filter (Amicon Ultra 3000 NMWL; Millipore) to remove the enzyme and stop the reaction. When NADH was added to the FMN‐bound yellowish indigo reductase solution, FMN was rapidly reduced to FMNH_2_ and the solution became colorless. When FMNH_2_ was rapidly oxidized, the solution turned yellowish again. The filtrate was then lyophilized twice using 99.9% D_2_O (Sigma‐Aldrich, St. Louis, MO, USA) [14]. As a control experiment, we used unlabeled NADH instead of deuterium‐labeled NADH. All samples examined by ^1^H‐NMR spectroscopy were dissolved in 0.6 mL of 99.9% D_2_O. Spectroscopic analysis was carried out using a Bruker Avance III 500 MHz spectrometer, with D_2_O (4.7 p.p.m.) as the reference. The data were processed using topspin 3.6.2 software (Bruker, Bremen, Germany).

### Docking simulation

Molecular docking simulations and binding energy calculations were performed using the autodock vina program [15]. autodock vina can produce a maximum of nine binding modes in each docking run. The hydrogen‐containing model structure of FMN bound to the indigo reductase dimer (chain B and C) of *B. smithii* and the molecular structure (PDBQT file format) of NAD^+^ were created using AutoDock tools. The area used to calculate docking was as follows: center *x* = −27.53, center *y* = 17.18, center *z* = 76.24, size *x* = 16.78, size *y* = 15.35, and size *z* = 19.40 Å, for indigo reductase (PDB entry 6JXN). Molecular graphics were created using pymol ver. 2.3.4 (https://pymol.org/2/).

## Results and Discussion

### Stereospecificity of hydrogen transfer from NADH to FMN

**Fig. 1:**
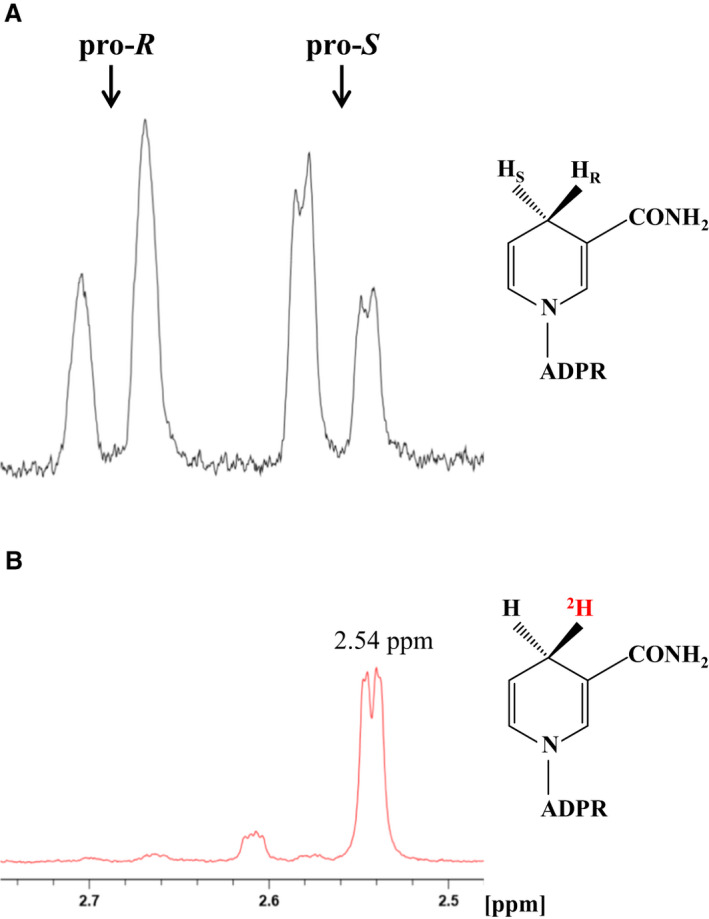
^1^H‐NMR spectra of the C4 position on the nicotinamide ring of NADH. (A) Standard unlabeled NADH. (B) 4*R*‐^2^H‐NADH produced from deuterated ethanol‐d_6_ (CD_3_CD_2_OD) and NAD^+^ using pro*‐R* stereospecific alcohol dehydrogenase. The arrows indicate the positions of the pro‐*S* and pro‐*R* proton signals.

**Fig. 2:**
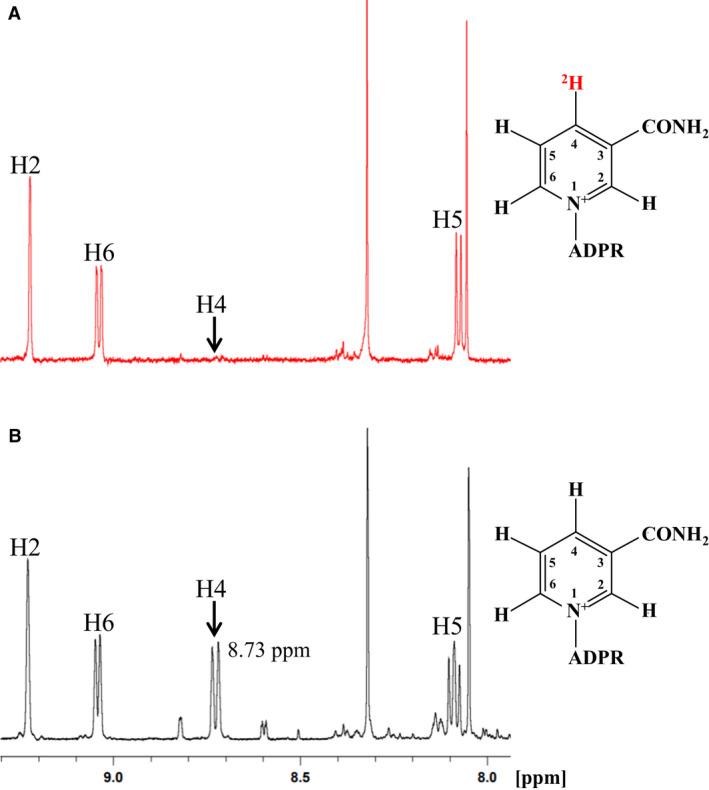
Position of the ^1^H‐NMR spectra for NAD^+^ produced by the *Bacillus smithii* indigo reductase with (A) 4*R*‐^2^H‐NADH and (B) unlabeled NADH. Labelled peaks are relative to the protons of the pyridine ring. Signals at 8.05 and 8.32 p.p.m. correspond to the protons of the adenosine moiety.

The two diastereotopic pro‐*R* and pro‐*S* hydrogen atoms at the C4 position of the nicotinamide ring of NADH were detected at 2.70 and 2.54 p.p.m. in the ^1^H‐NMR spectrum (Fig. 1A). However, in the ^1^H‐NMR spectrum of 4*R*‐^2^H‐NADH, the doublet signal at 2.70 p.p.m. disappeared (Fig. 1B) [16]. We determined the stereospecificity of the hydrogen transfer from NADH to FMN catalyzed by the *B. smithii* indigo reductase using 4*R*‐^2^H‐NADH. When 4*R*‐^2^H‐NADH was oxidized by the indigo reductase, a doublet signal from the C4 proton of NAD^+^ at around 8.73 p.p.m. was not observed in the ^1^H‐NMR spectrum (Fig. 2A). This indicates that the 4*R*‐^2^H of NADH remained in NAD^+^, and the 4*S*‐^1^H was removed and transferred to FMN. When NADH was used instead of ^2^H‐labeled NADH as the control, a resonance signal at around 8.73 p.p.m., reflecting the C4 proton of NAD^+^, was observed in the ^1^H‐NMR spectrum (Fig. 2B). Additionally, we confirmed a change in multiplicity of the signal of the H5 proton in the nicotinamide ring. These results indicate that *B. smithii* indigo reductase exhibits pro‐*S* specificity (B‐type stereospecificity) for hydrogen transfer from NADH.

### Possible NAD^+^ and FMN binding structure of *B. smithii* indigo reductase

**Fig. 3:**
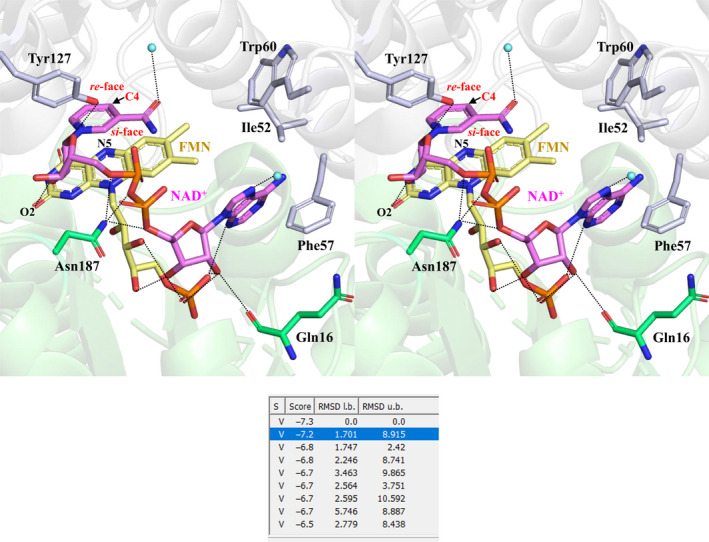
Molecular docking simulation predicted using the NAD^+^‐FMN binding model of *Bacillus smithii* indigo reductase plotted with the wall‐eyed stereo view. Residues that interact with NAD^+^ are labeled; gray: adjacent subunit. NAD^+^ (magenta) and FMN (yellow) are shown as stick models, water molecules (cyan) are shown as sphere models, and the network of hydrogen bonds is represented by black dotted lines. The C4 atom of the pyridine ring (a hydride acceptor site) and the *si*‐ and *re*‐faces are labeled. Oxygen, nitrogen, and phosphorus atoms are shown in red, blue, and orange, respectively. The values of binding energy (Score; kcal·mol^−1^) calculated by molecular docking simulations are shown in the table.

We performed molecular docking simulation using the AutoDock Vina program and modeled the NAD^+^ molecule at the active site of *B. smithii* indigo reductase (Fig. 3). The calculated binding energy between FMN‐bound indigo reductase and NAD^+^ was −7.2 kcal·mol^−1^. In our molecular docking simulation model, the oxygen atom in the amide group of the nicotinamide ring formed a hydrogen bond with a water molecule (3.4 Å). The nicotinamide ribose interacted with the side chain of Tyr127 (2.4 Å), which has been previously suggested to be the binding residue of indigo reductase substrate, and the O_2_ atom of the flavin ring (3.3 Å) in FMN. The phosphate group of NAD^+^ interacted with the side chains of Asn187 (3.0–3.4 Å). Further, the adenine ribose formed hydrogen bonds with the OH groups of the ribityl chain (2.7–2.9 Å), the oxygen in the main chain of Gln16 (3.1 Å), and the phosphate group of FMN (2.8–3.2 Å). The N7 atom of the adenine ring was observed to interact with a water molecule (2.7 Å). Moreover, the adenine ring of NAD^+^ was covered by hydrophobic residues, such as Ile52, Phe57, and Trp60. As shown in Fig. 3, the pro‐*S* hydrogen (*si*‐face) located at the C4 position of the nicotinamide moiety of NAD^+^ was oriented toward the flavin‐N5 atom of FMN (3.6 Å). In addition, the glycosidic bond between the nicotinamide ring of NAD^+^ and its associated ribose moiety appeared in the *syn* conformation.

Generally, pro‐*S‐*specific enzymes are known to bind to NAD^+^ in the *syn* conformation and pro‐*R‐*specific enzymes in the *anti* conformation [8]. These results also support that *B. smithii* indigo reductase belongs to the group of pro‐*S*‐specific hydride transfer (B‐type stereospecificity) enzymes. Another example of a stereospecificity of the hydride transfer between NADH and FMN is exhibited by the *S. cerevisiae* FMN‐dependent NAD(P)H‐quinone oxidoreductase, which is reported to exhibit pro‐*S*‐specific stereospecificity that is the same as that exhibited by *B. smithii* indigo reductase [11]. In conclusion, to our knowledge, we are the first to report the stereospecificity of the hydride transfer of NADH in a reaction catalyzed by indigo reductase and provide the 3D structure of NAD^+^‐FMN‐bound indigo reductase.

## Conflict of interest

The authors declare no conflict of interest.

## Author contributions

KY, HS, and TO conceived the study, designed the experiments, and wrote the manuscript. KY performed the experiments and the molecular docking simulation, and analyzed the data. KY, HS, TA, and TO helped with the preparation of the manuscript.

## Data Availability

The structural data that support these findings are openly available in the wwPDB at https://doi.org/10.2210/pdb6JXN/pdb.
